# Detection and Molecular Identification of Eight *Candida* Species in Clinical Samples by Simplex PCR

**DOI:** 10.3390/microorganisms10020374

**Published:** 2022-02-05

**Authors:** Eduardo García-Salazar, Gustavo Acosta-Altamirano, Paola Betancourt-Cisneros, María del Rocío Reyes-Montes, Emmanuel Rosas-De-Paz, Esperanza Duarte-Escalante, Alma Rosa Sánchez-Conejo, Esther Ocharan Hernández, María Guadalupe Frías-De-León

**Affiliations:** 1 Unidad de Investigación, Hospital Regional de Alta Especialidad de Ixtapaluca, Carretera Federal México—Puebla Km. 34.5, Pueblo de Zoquiapan, Ixtapaluca 56530, Mexico; eduardogs_01@hotmail.com (E.G.-S.); mq9903@live.com.mx (G.A.-A.); 2 Programa de Maestría en Ciencias de la Salud, Escuela Superior de Medicina, Instituto Politécnico Nacional, Mexico City 07340, Mexico; estherocharan@hotmail.com; 3 Unidad de Investigación en Sistemática Vegetal y Suelo, Facultad de Estudios Superiores Zaragoza, Universidad Nacional Autónoma de México, Mexico City 04510, Mexico; paola14_02@hotmail.com; 4 Departamento de Microbiología y Parasitología, Facultad de Medicina, Universidad Nacional Autónoma de México, Mexico City 04510, Mexico; remoa@unam.mx (M.d.R.R.-M.); dupe@unam.mx (E.D.-E.); 5 Unidad de Microbiología, Universitat Rovira i Virgili, Carrer de l’Escorxador, s/n, 43003 Tarragona, Spain; emmrodepaz@gmail.com; 6 Dirección General, Hospital Regional de Alta Especialidad de Ixtapaluca, Carretera Federal México—Puebla Km. 34.5, Pueblo de Zoquiapan, Ixtapaluca 56530, Mexico; dra.asanchez10@gmail.com

**Keywords:** *Candida* spp., simplex PCR, diagnostic, molecular identification

## Abstract

Systemic candidiasis is a frequent opportunistic mycosis that can be life-threatening. Its main etiological agent is *Candida albicans*; however, the isolation of non-*albicans Candida* species has been increasing. Some of these species exhibit greater resistance to antifungals, so the rapid and specific identification of yeasts is crucial for a timely diagnosis and optimal treatment of patients. Multiple molecular assays have been developed, based mainly on polymerase chain reaction (PCR), showing high specificity and sensitivity to detect and identify *Candida* spp. Nevertheless, its application in diagnosis has been limited due to specialized infrastructure or methodological complexity. The objective of this study was to develop a PCR assay that detects and identifies some of the most common pathogenic *Candida* species and evaluate their diagnostic utility in blood samples and bronchial lavage. A pair of oligonucleotides was designed, CandF and CandR, based on sequence analysis of the 18S-ITS1-5.8S-ITS2-28S region of the rDNA of *Candida* spp., deposited in GenBank. The designed oligonucleotides identified *C. albicans*, *C. glabrata*, *C. tropicalis*, *C. parapsilosis*, *C. krusei*/*Pichia kudriazevii*, *C. guilliermondii*/*Meyerozyma guilliermondii*, *C. lusitaniae*/*Clavispora lusitaniae*, and *C. dubliniensis* using simplex PCR based on the amplicon size, showing a detection limit of 10 pg/μL of DNA or 10^3^ yeasts/mL. Based on cultures as the gold standard, it was determined that the sensitivity (73.9%), specificity (96.3%), and the positive (94.4%) and negative (81.2%) predictive values of the PCR assay with the designed oligonucleotides justify their reliable use in diagnosis.

## 1. Introduction

In recent decades, the frequency of opportunistic yeast infections has been increasing, particularly in intensive care units [1]. This event is associated with the increase of the population with immunosuppression resulting from various diseases, such as cancer or HIV-AIDS, use of broad-spectrum antibiotics, organ transplantation, among others [1,2,3,4]. The most frequent opportunistic fungal infection is candidiasis, caused by yeasts of the genus *Candida,* which includes more than 200 species; however, only about 10% are recognized as pathogenic to humans and some animals [1,5]. These fungi can cause superficial infections that affect the skin or mucous membranes and systemic infections that can spread and be life-threatening [6]. The main etiological agent is *Candida albicans*; yet, the isolation of non-*albicans Candida* species like the *C. glabrata* complex, *C. tropicalis*, the *C. guilliermondii*/*Meyerozyma guilliermondii* complex, *C. dubliniensis*, the *C. parapsilosis* complex, *C. tropicalis*, and *C. krusei* is becoming more frequent [7,8,9,10,11,12]. The frequency with which each of the non-*albicans* species occurs varies between geographical regions. Nonetheless, it has been reported that among the most common species are *C. albicans*, *C. glabrata* complex, *C. tropicalis*, *C. parapsilosis* complex, *C. krusei*/*Pichia kudriazevii*, *C. guilliermondii*/*M. guilliermondii* complex, *C. dubliniensis*, *C. lusitaniae*/*Clavispora lusitaniae*, and *C. famata*/*Debaryomyces hansenii* [6,11]. Compared to *C. albicans*, some of these species are resistant to antifungal agents, such as *C. glabrata* and *C. krusei*/*P. kudriazevii*, which have greater resistance to fluconazole. In addition, *C. glabrata* has developed resistance to other azoles and echinocandins [13,14]. Different antifungal susceptibility profiles and different virulence attributes have been demonstrated among species, even among the members of *C. glabrata*, *C. guilliermondii*/*M. guilliermondii*, and *C. parapsilosis* complexes [15,16,17,18,19]. Since the pattern of antifungal susceptibility between *Candida* species may be different, the rapid identification of yeasts at the species level is crucial for timely diagnosis and optimal antifungal treatment. Therefore, for diagnostic purposes, multiple molecular assays have been developed, such as the polymerase chain reaction (PCR) in different formats (simplex, multiplex, nested, semi-nested, real-time, linked to enzyme immunoassay (PCR-EIA) or enzymatic restriction (PCR-RFLP)), peptide nucleic acid fluorescent in situ hybridization (PNA-FISH) and matrix-assisted laser desorption/ionization time-of-flight mass spectrometry (MALDI-TOF MS) [19,20,21]. The above assays have shown rapidity, high specificity, and sensitivity in detecting and identifying *Candida* spp. However, its use in in-hospital laboratories has been limited due partly to the absence of specialized infrastructure required or the methodological complexity of some tests. So, it is necessary to develop new assays to correctly identify *Candida* species using specific, simple, affordable, and accessible methodologies for any laboratory.

The objective of this study was to develop a PCR assay that detects and identifies in a specific and rapid manner some of the most common pathogenic *Candida* species and evaluate its diagnostic usefulness in blood samples and bronchoalveolar lavage (BAL).

## 2. Materials and Methods

### 2.1. Candida spp. Reference Strains and Clinical Isolates

Eight reference strains (*C. albicans* ATCC^®^ 18804™, *C. glabrata* ATCC^®^ 2001™, *C. tropicalis* ATCC^®^ 750™, *C. krusei* ATCC^®^ 6258™, *C. parapsilosis* ATCC^®^ 22019™, *C dubliniensis* MYA-646, *C. lusitaniae* ATCC^®^ 34449™ and *C. guilliermondii* ATCC^®^ 6260™), and 42 *Candida* spp. clinical isolates, previously identified by carbohydrate assimilation test, were used to test the specificity of the PCR assay with the designed oligonucleotides (Table 1). The clinical isolates were obtained from the fungi collection of the High Specialty Regional Hospital of Ixtapaluca (Hospital Regional de Alta Especialidad Ixtapaluca) Research Unit. All isolates and reference strains were grown on Sabouraud Dextrose Agar (Bioxon, Mexico City, Mexico) and stored at room temperature in a sterile isotonic saline solution.

### 2.2. Clinical Samples

For the evaluation of the diagnostic utility of the PCR assay, 50 clinical samples were included, of which 40 were whole blood and 10 were BAL, from patients who were requested culture for suspected fungal infection.

### 2.3. DNA Extraction from Yeasts

For DNA extraction, the reference strains and clinical isolates were cultured in 3 mL of YPD (1% yeast extract, 2% peptone, 2% dextrose) and incubated at 30 °C for 24 h. The yeasts were retrieved from the culture by centrifugation at 8000 rpm for 1 min, and the supernatant was discarded. The DNA from the yeasts was extracted using a Yeast DNA Preparation Kit (Jena Biosciences GmbH, GE, Jena, Germany), following the manufacturer’s instructions.

### 2.4. DNA Extraction from Clinical Samples

DNA from all samples was extracted using the QIAamp DNA mini kit (Qiagen, GE, Hilden, Germany). For quality control of the obtained DNAs, a fragment of the β-globin gene was amplified as described by Talamaci et al. [22].

### 2.5. Oligonucleotide Design

An exhaustive sequence search of the 18S-ITS1-5.8S-ITS2-28S regions of all *Candida* species deposited in the GenBank database was performed [23]. Subsequently, multiple alignment with the Clustal Omega program [24] was done, using these sequences. The alignment was used to design the oligonucleotides by searching for unique regions for each species, such as insertions, deletions of several nucleotides, or regions with unique sequences using the Bioedit ver 7.2.5 [25] and GeneDoc ver. 2.7 [26] programs. Once these regions were located, a pair of oligonucleotides was designed with the Primaclade program [27]. The PCR conditions using the designed oligonucleotides were established with the DNA of the reference strains.

### 2.6. Specificity Evaluation and Detection Limit of the PCR Assay

The specificity of the PCR assay with the designed oligonucleotides was analyzed using 30 DNAs from *Candida* spp. isolates, as well as DNA from other pathogenic fungi (*Histoplasma capsulatum*, *Sporothrix schenckii*, *Paracoccidioides brasiliensis*, *Cryptococcus neoformans*, *Aspergillus fumigatus*).

To determine the assay’s detection limit, 500-μL blood aliquots obtained from healthy and immunocompetent donors were used, which were inoculated with a yeast suspension to obtain 106, 105, 104, 103, 102, 101, and 100 yeasts/μL. Separately, 500-μL blood aliquots were inoculated with *C. albicans* ATCC^®^ 18804™ DNA to obtain concentrations from 20 ng/μL to 10 ag/μL. DNA from all samples was extracted using a QIAamp DNA mini kit (Qiagen, GE, Hilden, Germany) following the manufacturer’s instructions. PCR assays were performed with the amplification conditions established for the designed oligonucleotides and using 6 μL of DNA.

### 2.7. Evaluation of the Diagnostic Utility of the PCR Assay

The DNA obtained from 50 clinical samples (40 whole blood and 10 bronchial lavages) from patients diagnosed with a fever of unknown origin or pneumonia was amplified to evaluate the diagnostic utility of the PCR assay. The parameters of sensitivity (SE), specificity (SP), and positive and negative predictive values (PPV and NPV) were determined considering the result of the gold standard (culture).

## 3. Results

### 3.1. Oligonucleotides Design

**Figure 1:**
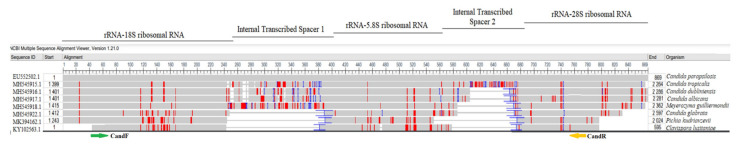
Multiple sequence alignment of region 18S-ITS1-5.8S-ITS2-28S of the eight *Candida* species identified by the oligonucleotides CandF and CandR. Blue lines indicate insertions, gray lines indicate deletions, and red lines indicate nucleotide changes. The pairing site of the CandF and CandR oligonucleotides is indicated by the green and yellow arrows, respectively. The image was generated through the MSA Viewer ver. 1.21.0 (https://blast.ncbi.nlm.nih.gov, accessed on 31 December 2021).

Five sequences of different *Candida* species were selected from the GenBank or, when applicable, all sequences found when the number was less than five for a species (Table 2). A pair of primers were designed, CandF and CandR, based on the analysis of unique regions for each species, such as insertions, deletions of several nucleotides, or regions with unique sequences (Figure 1). The sequences and characteristics of the primers are shown in Table 3.

**Figure 2:**
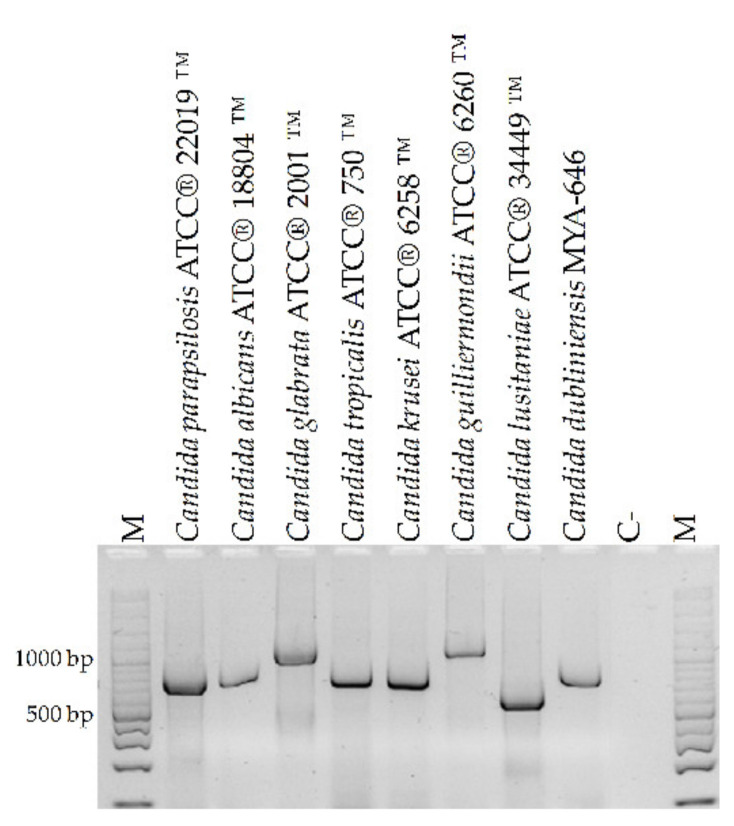
Amplification of DNA obtained from reference strains of *Candida* spp. using oligonucleotides CandF and CandR. M: 100 bp molecular size marker. C-: negative control (water).

CandF and CandR oligonucleotides amplified fragments of the expected size for each species when the DNA of the reference strains was amplified (Figure 2).

### 3.2. Specificity Evaluation and Detection Limit of the PCR Assay

**Figure 3:**
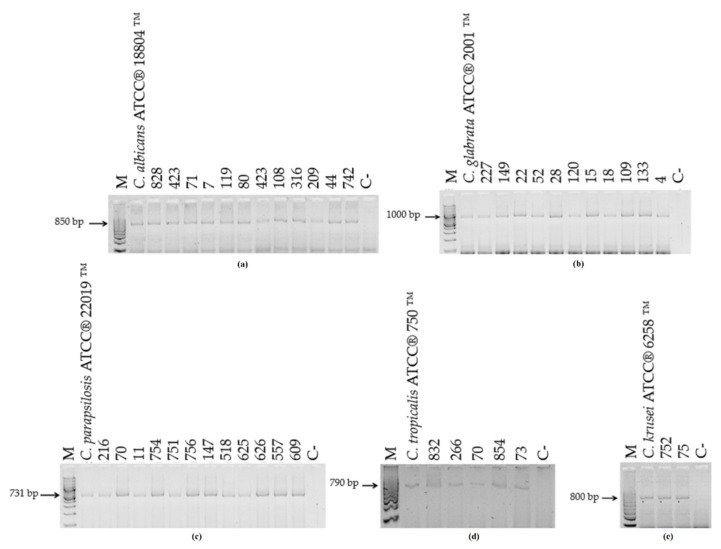
Specificity of the PCR assay with CandF and CandR oligonucleotides using DNA from clinical isolates of *Candida* spp. Amplification of DNA from different isolates of (**a**) *Candida albicans* isolates; (**b**) *Candida glabrata* isolates; (**c**) *Candida parapsilosis* isolates; (**d**) *Candida tropicalis* isolates; and (**e**) *Candida krusei* isolates. M—100-bp molecular size marker; C-—negative control (water).

**Figure 4:**
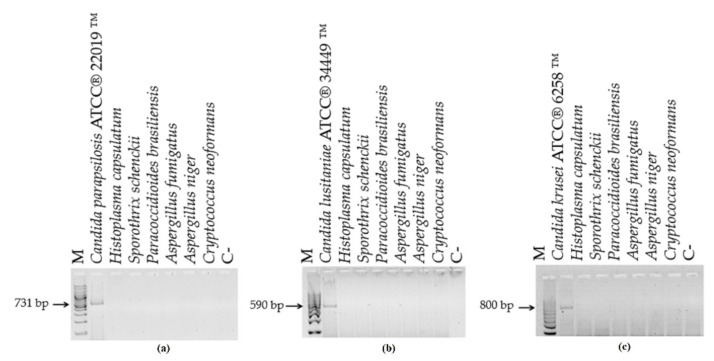
Specificity of the PCR assay with CandF and CandR oligonucleotides using DNA from other pathogenic fungi. DNA from other fungi did not amplify the specific fragments of (**a**) *Candida parapsilosis*; (**b**) *Candida lusitaniae* or (**c**) *Candida krusei*. M—100-bp molecular size marker; C-—negative control (water).

The DNA analysis of the 42 clinical isolates showed concordance between PCR-generated amplicon and biochemical identification results (Figure 3). No amplification was observed when the DNA from *H. capsulatum*, *S. schenckii*, *P. brasiliensis*, *C. neoformans*, or *A. fumigatus* were amplified (Figure 4).

**Figure 5:**
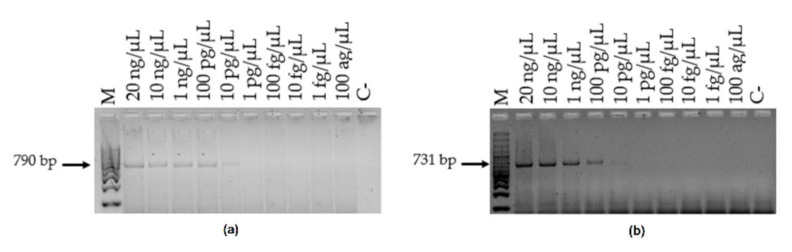
Detection limit of the PCR assay using CandF and CandR oligonucleotides. (**a**) Amplification of different concentrations of *Candida tropicalis* ATCC^®^ 750™ DNA; (**b**) Amplification of different concentrations of *Candida parapsilosis* ATCC^®^ 22019™ DNA. M: 100 bp molecular size marker. C-—negative control (water).

Amplification of different DNA concentrations showed a detection limit of 10 pg/μL, as amplicons were detected from a concentration of 20 ng/μL to 10 pg/μL. However, no amplification was observed at lower concentrations (Figure 5). The same results were noted with the DNA from the eight *Candida* species identified by oligonucleotides. The detection limit using different yeast concentrations was 10^3^ yeasts/mL for each species since the amplicon was observed between 10^6^ and 10^3^ yeasts/mL but was no longer viewed from 10^2^ yeasts/mL up to 10^0^.

### 3.3. Evaluation of the Diagnostic Utility of the PCR Assay

Based on the concordance between the results obtained by PCR and the results from the gold standard (Table 4 and Table 5), six (12%) false-negative results and one (2%) false-positive result were found. It was determined that the PCR test with the designed oligonucleotides had a sensitivity of 73.9%, a specificity of 96.3%, and positive and negative predictive values of 94.4% and 81.2%, respectively.

## 4. Discussion

Candidiasis is one of the most common fungal infections in the immunosuppressed population. Its diagnosis must be accurate and rapid to allow the establishment of timely and specific treatment, especially in systemic or invasive infections, in which life can be compromised [6]. Cultures are considered the gold standard for diagnosing candidiasis; however, they present several limitations that hinder the proper management of patients. Among these limitations, it must be mentioned that the sensitivity is relatively low, particularly in blood samples where the negativity percentage is close to 50%. Additionally, the isolation of yeasts through culture in bronchial lavages is not that simple. Furthermore, about 19% of false negatives have been reported in bronchial lavage [28,29]. Another limitation of the gold standard is the time required to obtain results, requiring at least three days for isolation plus additional days for the species identification (48–96 h) [29]. That is why molecular methods have gained importance. Even though diverse and novel procedures have been developed, with various degrees of sensitivity and specificity, their use in practice has been limited by the need for infrastructure and highly specialized personnel. Considering the prior, we have designed a pair of primers (CandF and CandR) that detect and identify the presence of eight of the most common *Candida* species causing disease by simplex end-point PCR, without the need to perform amplicon sequencing to define the species. CandF and CandR were designed based on the 18S-ITS1-5.8S-ITS2-28S region, taking advantage of the ITS regions intraspecific variability and the conserved sequences of the 18S, 5.8S, and 28S regions. In this way, eight of the most frequent pathogenic species were differentiated (*C. albicans*, *C. glabrata*, *C. tropicalis*, *C. parapsilosis*, *C. krusei*/*P. kudriazevii*, *C. guilliermondii*/*M. guilliermondii*, *C. lusitaniae*/*C. lusitaniae*, and *C. dubliniensis*) based on the amplicon size, avoiding the sequencing step that is used in other PCR assays that employ this genomic region as a basis [30]. The oligonucleotides CandF and CandR proved to be species-specific. They only amplified the DNA of any of the eight *Candida* species for which they were designed and did not amplify the genetic material of other fungal pathogens or human DNA. The detection limit of the designed oligonucleotides was 10 pg/μL, which is comparable to that of other trials reported in the literature [30,31,32,33].

The parameters that determine the diagnostic usefulness of the PCR with the CandF and CandR oligonucleotides were adequate. The sensitivity found (73.9%) indicates that less than three out of ten patients with candidiasis will not be detected. On the other hand, 96.3% of patients who do not suffer from candidiasis will have a negative result when the PCR is used with CandF and CandR oligonucleotides. The PPV found (94.4%) indicates that a PCR test with the designed oligonucleotides has a high probability of association with candidiasis. At the same time, the NPV (81.2%) shows that 81.2% of patients with a negative PCR will have a negative culture. These results confirm that a PCR with the designed oligonucleotides can be used to diagnose patients with fever of unknown origin who do not respond to antibacterial treatment. Nevertheless, it is essential to mention that this PCR assay has both advantages and limitations, as with any other assay. Among the advantages, we can remark the rapid identification of eight of the most frequent pathogenic species, without the need to perform additional procedures to the amplification, such as sequencing. Another advantage is that the infrastructure needed to implement this simplex end-point PCR does not require much highly specialized equipment or infrastructure, so it can be easily implemented in any laboratory with the minimum tools to perform a PCR assay. Likewise, the cost of each assay is lower compared to other methods, such as real-time PCR. One additional advantage is the time reduction compared to the completion of a culture and the subsequent identification of the isolated yeast. In a single step, the PCR with CandF and CandR detects and identifies the yeast at the species level. It is worth noting that in this study, the species identification was consistent among PCR and Vitek 2, which is the automated biochemical system used to identify yeasts after their isolation in cultures.

Among the disadvantages, we can mention that how amplicons are visualized (UV light) can lead to false-negative results, as low concentrations of template DNA can lead to little or no visible amplification products. Another limitation is that CandF and CandR oligonucleotides only identify eight of the more than 20 species known to cause infection in humans, including *C. auris*, a yeast that has emerged globally as a multidrug-resistant pathogen associated with healthcare [34]. Therefore, if the etiological agent is another species, it cannot be identified, leading to false-negative results. The latter occurred with sample 33, which did not amplify with the designed oligonucleotides but gave an isolated yeast biochemically identified as *C. famata* by conventional methods (culture and biochemical identification). Besides, another restraint is that the CandF and CandR oligonucleotides recognize *C. glabrata*, *C. parapsilosis*, and *C. guilliermondii*/*M. guilliermondii* only at the complex level, which means that it is not possible to differentiate between *C. glabrata sensu stricto*, *C. bracarensis*, and *C. nivariensis*. Fortunately, the frequency of *C. bracarensis* and *C. nivariensis*, as well as the species that compose the *C. parapsilosis* and *C. guilliermondii*/*M. guilliermondii* complexes, is low [35,36,37].

## 5. Conclusions

The CandF and CandR oligonucleotides specifically identify eight of the most frequent Candida species that cause infection (*C. albicans*, *C. glabrata*, *C. tropicalis*, *C. parapsilosis*, *C. krusei*/*P. kudriazevii*, *C. guilliermondii*/*M. guilliermondii*, *C. lusitaniae*/*C. lusitaniae*, and *C. dubliniensis*) with a detection limit of 10 pg/μL of DNA or 10^3^ yeasts/mL. In addition, the PCR assay with the CandF and CandR primers showed a sensitivity, specificity, PPV, and NPV that justify their reliable use in diagnosing systemic candidiasis.

## Figures and Tables

**Table 1:** Clinical isolates of *Candida* spp. used in this study.

Isolation	Species
828	*Candida albicans*
423	*Candida albicans*
71	*Candida albicans*
7	*Candida albicans*
119	*Candida albicans*
80	*Candida albicans*
423	*Candida albicans*
108	*Candida albicans*
316	*Candida albicans*
209	*Candida albicans*
44	*Candida albicans*
742	*Candida albicans*
227	*Candida glabrata*
149	*Candida glabrata*
22	*Candida glabrata*
52	*Candida glabrata*
28	*Candida glabrata*
120	*Candida glabrata*
15	*Candida glabrata*
18	*Candida glabrata*
109	*Candida glabrata*
133	*Candida glabrata*
4	*Candida glabrata*
216	*Candida parapsilosis*
70	*Candida parapsilosis*
11	*Candida parapsilosis*
754	*Candida parapsilosis*
751	*Candida parapsilosis*
756	*Candida parapsilosis*
147	*Candida parapsilosis*
518	*Candida parapsilosis*
625	*Candida parapsilosis*
626	*Candida parapsilosis*
557	*Candida parapsilosis*
609	*Candida parapsilosis*
832	*Candida tropicalis*
266	*Candida tropicalis*
70	*Candida tropicalis*
854	*Candida tropicalis*
73	*Candida tropicalis*
752	*Candida krusei*/*Pichia kudriazevii*
75	*Candida krusei*/*Pichia kudriazevii*

**Table 2:** Sequences of the 18S-ITS1-5.8S-ITS2-28S region of *Candida* spp. used for multiple alignment and oligonucleotide design.

Species	GenBank Access Number
*C. albicans*	KF241849.1, KF241847.1, MH545917.1, KC905077.1, KC905075.1, KC905076.1
*C. glabrata sensu stricto*	KP675655.1, KP068740.1, KP675693.1, MH545922.1, KC253980.1, JN391276.1
*C. nivariensis*	KP068747.1, KP068746.1, KP131740.1
*C. bracarensis*	JN882338.1, KP674833.1, JN882340.1, GU199439.1
*C. tropicalis*	JF709971.1, MH545915.1, JQ008834.1, EU589204.1
*C. parapsilosis sensu stricto*	EU552502.1, EU564205.1, EU552500.1, EU564204.1
*C. metapsilosis*	EU484055.1, KM014585.1, EU564207.1
*C. orthopsilosis*	EU557370.1, EU557371.1, EU552495.1, EU557373.1, EU557372.1
*C. krusei*/*P. kudriazevii*	KC886644.1, MK394162.1, KF959839.1, KF959838.1
*C. guilliermondii*/*M. guilliermondii sensu stricto*	U45709.1, MH545918.1
*C. fermentati*	AY187283.1
*C. carpophila*/*C. xestobii*	U45707.1
*C. smithsonii*	AY518525.1
*C. athensensis*	AY518525.1
*C. elateridarum*	AY518530.1
*C. lusitaniae*/*C. lusitaniae*	KP131851.1, KY102563.1, AY493434.1, KP131846.1, KP131844.1, KP131839.1
*C. kefyr*/*Kluyveromyces marxianus*	KC905771.1
*C. famata*/*D. hansenii*	GQ376085.1
*C. rugosa*	GU144663.1
*C. dubliniensis*	KP131696.1, KP131697.1, MH545916.1, KC905080.1, KC905078.1, KC905079.1
*C. norvegensis*	AB278166.1, AB278165.1, AB278169.1, AB278163.1, AB278162.1
*C. lipolytica*	KP132909.1, KP132908.1, KP132907.1
*C. sake*	FJ515167.1, KM384608.1, KM384082.1, KM384081.1
*C. pelliculosa*	KP132885.1, KP132887.1, KP132884.1
*C. apícola*	EU926481.1, EU926480.1, EU926479.1, EU926486.1
*C. zeylanoides*	HE799676.1, HE799675.1, AB278160.1
*C. valida*	KF057628.1, KF057621.1, KF057630.1, KF057617.1
*C. intermedia*	KP131722.1
*C. pulcherrima*	EF449525.1, AY301026.1
*C. haemulonii*	JX459675.1, JX459674.1, KJ706229.1, JX459661.1
*C. utilis*	KP132000.1, KP131999.1
*C. humícola*	HM459599.1, KC118118.1, GU256753.1, JN882338.1, KP674833.1, JN882340.1, GU199439.1, J515176.1
*C. lambica*	KF646205.1, KF646196.1, KP132501.1, KF646181.1
*C. ciferrii*	KP132796.1, KP132795.1
*C. colliculosa*	HE799671.1
*C. marina*	KJ707187.1, KJ706412.1, KJ707232.1, KJ707196.1
*C. sphaerica*	HE799667.1
*C. holmii*	KM374151.1

**Table 3:** Oligonucleotides designed for the identification of eight *Candida* species.

Oligonucleotide Name	Sequence (5′-3′)	Identified *Candida* Species	Amplicon Size (pb)
CandF	AGCTTGCGTTGATTACGTCCCTGCCC	*C. albicans*	850
		*C. glabrata*	1000
		*C. tropicalis*	790
		*C. parapsilosis*	731
CandR	TTCACTCGCCGCTACTAAGGCAATCCC	*C. krusei*/*P. kudriazevii*	800
		*C. guilliermondii*/*M. guilliermondii*	1100
		*C. lusitaniae*/*C. lusitaniae*	590
		*C. dubliniensis*	810

**Table 4:** Detection and identification of *Candida* spp. in blood and BAL samples.

Sample Number	Type of Sample	PCR with Cand Primers			Culture	
		Positive (P)/Negative (N)	Amplicon Size (bp)	Species	Positive (P)/Negative (N)	Species *
1	blood	N			N	
2	blood	N			P	*C. albicans*
3	blood	N			N	
4	blood	P	731	*C. parapsilosis*	P	*C. parapsilosis*
5	blood	P	731	*C. parapsilosis*	P	*C. parapsilosis*
6	blood	P	850	*C. albicans*	N	
7	blood	N			N	
8	blood	P	850	*C. albicans*	P	*C. albicans*
9	blood	N			N	
10	blood	N			N	
11	blood	N			N	
12	blood	N			P	*C. glabrata*
13	blood	P	850	*C. albicans*	P	*C. albicans*
14	blood	P	850	*C. albicans*	P	*C. albicans*
15	blood	P	731	*C. parapsilosis*	P	*C. parapsilosis*
16	blood	P	850	*C. albicans*	P	*C. albicans*
17	blood	N			N	
18	blood	P	850	*C. albicans*	P	*C. albicans*
19	blood	N			N	
20	blood	N			N	
21	blood	N			P	*C. parapsilosis*
22	blood	P	731	*C. parapsilosis*	P	*C. parapsilosis*
23	blood	N			N	
24	blood	P	790	*C. tropicalis*	P	*C. tropicalis*
25	blood	N			N	
26	blood	N			N	
27	blood	N			N	
28	blood	P	850	*C. albicans*	P	*C. albicans*
29	blood	N			N	
30	blood	P	850	*C. albicans*	P	*C. albicans*
31	blood	N			N	
32	blood	P	850	*C. albicans*	P	*C. albicans*
33	blood	N			P	*C. famata*/*D. hansenii*
34	blood	P	850	*C. albicans*	P	*C. albicans*
35	blood	P	731	*C. parapsilosis*	P	*C. parapsilosis*
36	blood	N			N	
37	blood	N			P	*C. albicans*
38	blood	N			N	
39	blood	P	1000	*C. glabrata*	P	*C. glabrata*
40	blood	N			N	
41	BAL	N			N	
42	BAL	N			N	
43	BAL	P	850	*C. albicans*	P	*C. albicans*
44	BAL	N			N	
45	BAL	N			N	
46	BAL	N			N	
47	BAL	N			N	
48	BAL	N			P	*C. albicans*
49	BAL	N			N	
50	BAL	N			N	

BAL: Bronchoalveolar lavage; * All growth-positive cultures were tested by a commercial Vitek 2 yeast identification system.

**Table 5:** PCR test with CandF and CandR oligonucleotides compared to gold standard (culture).

		**Gold Standard (Culture)**		
PCR with Cand primers		**Present Disease**	**Absent Disease**	**Total**
	Positive PCR	true positives: 17	false positives: 1	individuals with positive test: 18
	Negative PCR	false negatives: 6	true negatives: 26	individuals with negative test: 32
	Total	sick individuals: 23	non-sick individuals: 27	individuals included: 50

## References

[1] Gnat S., Łagowski D., Nowakiewicz A., Dyląg M. (2021). A global view on fungal infections in humans and animals: Opportunistic infections and microsporidioses. J. Appl. Microbiol..

[2] Garbee D.D., Pierce S.S., Manning J. (2017). Opportunistic Fungal infections in critical care units. Crit. Care Nurs. Clin. N. Am..

[3] De Oliveira Santos G.C., Vasconcelos C.C., Lopes A.J.O., de Sousa Cartágenes M.D.S., Filho A.K.D.B., do Nascimento F.R.F., Ramos R.M., Pires E.R.R.B., de Andrade M.S., Rocha F.M.G. (2018). Candida infections and therapeutic strategies: Mechanisms of action for traditional and alternative agents. Front. Microbiol..

[4] Jenks J.D., Cornely O.A., Chen S.C., Thompson G.R., Hoenigl M. (2020). Breakthrough invasive fungal infections: Who is at risk?. Mycoses.

[5] Pal M., Gebrezgabher W., Samajpati N., Manna A.K. (2015). Growing role of non-Candida albicans species in clinical disorders of human and animals. J. Mycopathol. Res..

[6] Pappas P., Lionakis M., Arendrup M., Ostrosky-Zeichner L., Kullberg B.J. (2018). Invasive candidiasis. Nat. Rev. Dis. Primers.

[7] Bassetti M., Righi E., Costa A., Fasce R., Molinari M.P., Rosso R., Pallavicini F.B., Viscoli C. (2006). Epidemiological trends in nosocomial candidemia in intensive care. BMC Infect. Dis..

[8] Bassetti M., Giacobbe D.R., Vena A., Wolff M. (2019). Diagnosis and treatment of candidemia in the intensive care unit. Semin. Respir. Crit. Care Med..

[9] Pfaller M.A., Jones R.N., Castanheira M. (2014). Regional data analysis of Candida non-albicans strains collected in United States medical sites over a 6-year period, 2006–2011. Mycoses.

[10] Pfaller M.A., Diekema D.J., Turnidge J.D., Castanheira M., Jones R.N. (2019). Twenty Years of the SENTRY Antifungal Surveillance Program: Results for Candida Species From 1997–2016. Open Forum Infect. Dis..

[11] Reyes-Montes M.D.R., Duarte-Escalante E., Martínez-Herrera E., Acosta-Altamirano G., Frías-De León M.G. (2017). Current status of the etiology of candidiasis in Mexico. Rev. Iberoam. Micol..

[12] Lamoth F., Lockhart S.R., Berkow E.L., Calandra T. (2018). Changes in the epidemiological landscape of invasive candidiasis. J. Antimicrob. Chemother..

[13] Rivero-Menendez O., Navarro-Rodriguez P., Bernal-Martinez L., Martin-Cano G., Lopez-Perez L., Sanchez-Romero I., Perez-Ayala A., Capilla J., Zaragoza O., Alastruey-Izquierdo A. (2019). Clinical and laboratory development of echinocandin resistance in Candida glabrata: Molecular characterization. Front. Microbiol..

[14] Pristov K.E., Ghannoum M.A. (2019). Resistance of Candida to azoles and echinocandins worldwide. Clin. Microbiol. Infect..

[15] Tosun I., Akyuz Z., Guler N.C., Gulmez D., Bayramoglu G., Kaklikkaya N., Arikan-Akdagli S., Aydin F. (2013). Distribution, virulence attributes and antifungal susceptibility patterns of Candida parapsilosis complex strains isolated from clinical samples. Med. Mycol..

[16] Neji S., Hadrich I., Trabelsi H., Abbes S., Cheikhrouhou F., Sellami H., Makni F., Ayadi A. (2017). Virulence factors, antifungal susceptibility and molecular mechanisms of azole resistance among Candida parapsilosis complex isolates recovered from clinical specimens. J. Biomed. Sci..

[17] Cheng J.W., Yu S.Y., Xiao M., Wang H., Kudinha T., Kong F., Xu Y.C. (2016). Identification and Antifungal Susceptibility Profile of *Candida guilliermondii* and *Candida fermentati* from a Multicenter Study in China. J. Clin. Microbiol..

[18] Hou X., Xiao M., Chen S.C.A., Kong F., Wang H., Chu Y.Z., Kang M., Sun Z.Y., Hu Z.D., Li R.Y. (2017). Molecular Epidemiology and Antifungal Susceptibility of Candida glabrata in China (August 2009 to July 2014): A Multi-Center Study. Front. Microbiol..

[19] Kim H.J., Brehm-Stecher B.F. (2015). Design and evaluation of peptide nucleic acid probes for specific identification of Candida albicans. J. Clin. Microbiol..

[20] Turhan O., Ozhak-Baysan B., Zaragoza O., Er H., Eres Sarıtas Z., Ongut G., Ogunc D., Colak D., Cuenca-Estrella M. (2017). Evaluation of MALDI-TOF-MS for the identification of yeast isolates causing bloodstream infection. Clin. Lab..

[21] Fidler G., Leiter E., Kocsube S., Biro S., Paholcsek M. (2018). Validation of a simplex PCR assay enabling reliable identification of clinically relevant Candida species. BMC Infect. Dis..

[22] Talmaci R., Traeger-Synodinos J., Kanavakis E., Coriu D., Colita D., Gavrila L. (2004). Scanning of beta-globin gene for identification of beta-thalassemia mutation in Romanian population. J. Cell Mol. Med..

[23] NCBI An Online Data Base Currently Coordinated and Supported by National Center for Biothecnology. http://ncbi.nlm.nih.gov.

[24] Clustal Ω Multiple Sequence Alignment. http://www.ebi.ac.uk/Tools/msa/clustalo/.

[25] Hall T.A. (1999). BioEdit: A user-friendly biological sequence alignment editor and analysis program for Windows 95/98/NT. Nucl. Acids Symp. Ser..

[26] Nicholas K.B., Nicholas H.B., Deerfield D.W. (1997). GeneDoc: Analysis and Visualization of Genetic Variation. EMBnet.news.

[27] Michael D.G., Simon T.M., Andrew N.D., Elizabeth A. (2005). Primaclade a flexible tool to find conserved PCR primers across multiple species. Bioinformatics.

[28] Schabereiter-Gurtner C., Selitsch B., Rotter M.L., Hirschl A.M., Willinger B. (2007). Development of novel real-time PCR assays for detection and differentiation of eleven medically important Aspergillus and Candida species in clinical specimens. J. Clin. Microbiol..

[29] Taira C.L., Okay T.S., Delgado A.F., Rivero Ceccon M.E.J., Gottardo de Almeida M.G., Barbaro Del Negro G.M. (2014). A multiplex nested PCR for the detection and identification of Candida species in blood samples of critically ill paediatric patients. BMC Infect. Dis..

[30] Mohammadi R., Abdi S. (2015). Molecular identification of Candida species isolated from gastro-oesophageal candidiasis in Tehran, Iran. Gastroenterol. Hepatol. Bed Bench.

[31] Chang H., Leaw S., Huang A., Wu T., Chang T. (2001). Rapid identification of yeasts in positive blood cultures by a Multiplex PCR method. J. Clin. Microbiol..

[32] Estrada-Barraza D., Dávalos M.A., Flores P.L., Mendoza D.R., Sánchez V.L. (2011). Comparación entre métodos convencionales, ChromAgar Candida y el método de la PCR para la identificación de especies de Candida en aislamientos clínicos. Rev. Iberoam. Micol..

[33] Merseguel K., Nishikaku A., Rodrigues A., Padovan A., e Ferreira R., Salles de Azevedo Melo A., Ribeiro da Silva Briones M., Lopes Colombo A. (2015). Genetic diversity of medically important and emerging Candida species causing invasive infection. BMC Infect. Dis..

[34] Du H., Bing J., Hu T., Ennis C.L., Nobile C.J., Huang G. (2020). Candida auris: Epidemiology, biology, antifungal resistance, and virulence. PLoS Pathog..

[35] Bonfietti L.X., Dos Anjos Martins M., Walderez Szeszs M., Stolf Pukiskas S.B., Ueda Purisco S., Cortez Pimentel F., Hanna Pereira G., Silva D.C., Oliveira L., de Souza Carvalho Melhem M. (2012). Prevalence, distribution and antifungal susceptibility profiles of *Candida parapsilosis*, *Candida orthopsilosis* and *Candida metapsilosis* bloodstream isolates. J. Med. Microbiol..

[36] Marcos-Zambrano L.J., Puig-Asensio M., Pérez-García F., Escribano P., Sánchez-Carrillo C., Zaragoza O., Padilla B., Cuenca-Estrella M., Almirante B., Martín-Gómez M.T. (2017). *Candida guilliermondii* Complex Is Characterized by High Antifungal Resistance but Low Mortality in 22 Cases of Candidemia. Antimicrob. Agents Chemother..

[37] Małek M., Mrowiec P., Klesiewicz K., Skiba-Kurek I., Szczepański A., Białecka J., Żak I., Bogusz B., Kędzierska J., Budak A. (2019). Prevalence of human pathogens of the clade Nakaseomyces in a culture collection—Tthe first report on Candida bracarensis in Poland. Folia Microbiol..

